# Different Breast Cancer Subtypes Show Different Metastatic Patterns: A Study from A Large Public Database

**DOI:** 10.31557/APJCP.2020.21.12.3587

**Published:** 2020-12

**Authors:** Yi Guo, Cletus A Arciero, Renjian Jiang, Madhusmita Behera, Limin Peng, Xiaoxian Li

**Affiliations:** 1 *Department of Biostatistics and Bioinformatics, Emory University, Atlanta, GA, USA. *; 2 *Department of Surgery, Emory University, Atlanta, GA, USA. *; 3 *Winship Cancer Institute, Emory University, Atlanta, GA, USA. *; 4 *Department of Pathology and Laboratory Medicine, Emory University, Atlanta, GA, USA. *

**Keywords:** Breast cancer, metastasis, bone metastasis, liver metastasis, brain metastasis, lung metastasis

## Abstract

**Background:**

Receptor status in breast cancer is known to be related to survival. However, the relationship between breast cancer subtype, preferential sites of metastasis, and overall survival is not clear.

**Methods:**

A total of 414,528 patients from the National Cancer Database (2010-2013) were examined. All patients received surgery and systemic treatments. Breast cancer was subtyped based on hormonal receptor (HR) and HER2 status.

**Results:**

HR-/HER2+ breast cancer patients had the highest overall rate of metastasis while HR+/HER2- had the lowest. HR+/HER2+ cancer had the most frequent metastasis to the bone, and HR-/HER2+ to brain, liver, lung and multiple sites. Generally, patients with brain or multiple metastasis had the worst overall survival (OS) across different subtypes. Patients with bone oligometastasis tend to have better OS than patients with metastasis to other site but significantly worse OS than patients without any metastasis.

**Conclusions:**

This large study exhibits how breast cancer subtype plays a role in the rate and site of metastasis as well as in overall survival. Surveillance and treatment strategies should be tailored on the risk and potential site of metastases based upon receptor subtype.

## Introduction

Breast cancer is the most common cancer in women worldwide and leads to over 40,000 deaths in the United States each year.(“Cancer STAT Facts: Female Breast Cancer,” 2017; Torre et al., 2015) These breast cancer related deaths are most often secondary to distant metastases, which are classified as either primary or secondary. Primary metastasis is referred as having distant metastasis at presentation and comprises 4.1-8.5% of patients presenting with breast cancer (Andre et al., 2004; Gong et al., 2017; Kast et al., 2015; Sant et al., 2004). Secondary metastasis occurs in patients who have already received definitive therapy, and can comprise up to 30% of breast cancer patients (Andre et al., 2004; Sant et al., 2004). Survival from either primary or secondary metastatic breast cancer is in part related to the receptor status of the tumor as well as the site of metastatic disease (Kast et al., 2015). Overall survival from breast cancer, even when metastatic, continues to improve (Chia et al., 2007). However, the identification of patients at highest risk for metastasis continues to be a challenge for clinicians. 

There has been extensive research examining factors related to metastasis, specifically examining the receptor status of a tumor both as a risk factor for metastasis and as a factor in the potential site of metastasis as well as overall survival (Falck et al., 2013; Kennecke et al., 2010). Studies have correlated estrogen receptor (ER), progesterone receptor (PR) and human epidermal growth factor receptor (HER2) status with metastasis and survival (Garcia Fernandez et al., 2012; Gerratana et al., 2015; Kast et al., 2015; Perou et al., 2000). Triple negative breast cancer (TNBC) and HER2 positive (HER2+) cancer have poorer overall survival and high rates of metastasis. Hormone receptor positive tumors (ER+ or PR+) have better overall survival and lower rates of metastasis.

Although these studies have shed light on the relationship between breast cancer subtype and metastasis, most studies have been on a relatively small scale. This study examined a large patient cohort from a national database to study the relationship between breast cancer subtype and site and rate of metastasis and overall survival in patients with secondary metastatic breast cancer.

## Materials and Methods

*Patients and Methods*

*Patient information*

We searched breast cancer patients diagnosed from 2010 to 2013 in the American College of Surgeon and American Cancer Society’s National Cancer Database (NCDB). All patients had surgery for the breast cancer. We excluded patients if not receiving any systemic treatment (endocrine therapy, chemotherapy or HER2 targeted therapy). A total of 414,528 patients were included. We collected the following information of the patients: age at diagnosis (age), status of hormonal receptor (estrogen receptor or progesterone receptor) and HER2, site of metastasis and overall survival (OS). Hormonal receptor (HR) was defined as positive when either ER or PR was positive. The breast cancer was classified by the HR and HER2 status into four subtypes: HR+/HER2–, HR+/HER2+, HR–/HER2+ and HR–/HER2–. The HR-/HER2- subtype was also referred as triple negative breast cancer (TNBC). 

*Statistical analysis*

Demographic and clinicopathological characteristics including patient age at diagnosis (defined as a binary variable: age ≤ 50 or > 50 years), chemotherapy, radiation therapy, hormonal therapy and HER2 targeted therapy were summarized by count and proportion. The association between metastatic sites and breast cancer subtypes was assessed by a Chi-square test. The OS times of patients were summarized using Kaplan-Meier survival curves and assessed by log-rank tests . Univariate Cox proportional hazards models were used to compare overall survival (OS) between patient groups with different sites of metastasis (e.g. bone, brain, lung, liver, multiple) or without metastasis stratified by breast cancer subtype. A p-value less than 0.05 was considered as statistically significant. All statistical analyses were two-tailed and conducted using SAS version 9.4 software (SAS Institute, Cary, NC).

## Results

*Patient demographic and clinical information*

The follow-up time was up to 71.98 months with a median of 36.37 months. Table 1 summarized the demographic and clinicopathological characteristics of the patients. Of the 414,528 patients, the majority of patients were older than 50 years (76.58%). All patients had surgery; 86.79% received hormonal therapy, 53.17% received chemotherapy and 2.83% received HER2 targeted therapy (Table 1). A total of 5,185 (1.25%) patients developed metastatic disease during the study period. The site of metastasis was most commonly bone (2,803, 54.06%) followed by lung (735, 14.18%), liver (544, 10.50%) and brain (83, 1.60%). There were 1,020 patients (19.67%) with multiple metastasis at discovery of distant disease.

Different breast cancer subtype had different metastatic pattern; HR-/HER2+ subtype had the most frequent metastasis to brain, liver, lung and multiple metastases. Table 2 summarized the metastatic sites of different breast subtypes. The HR-/HER2+ had the most frequent brain metastasis (0.1%) compared with 0.01% in HR+/HER2- breast cancer, 0.02% in HR+/HER2+ and 0.07% in TNBC (P < 0.0001). The HR-/HER2+ subtype also had the most frequent metastasis to the liver (0.9%) and the lung (0.55%) and the most frequent multiple metastasis (0.79%) compared with other subtypes including TNBC (P < 0.0001, Table 2). The HR+/HER2+ breast cancer had the most frequent bone metastasis (1.06%) and the TNBC had the least (0.52%) (P < 0.0001). Overall, HR-/HER2+ breast cancer had the most frequent rate (3.1%) of metastasis (combine bone, brain, liver, lung and multiple metastases) and the HR+/HER2- had the least (1%) (P < 0.0001). 

*Breast cancer patients with metastasis to different organs showed different overall survival*

**Figure 1:**
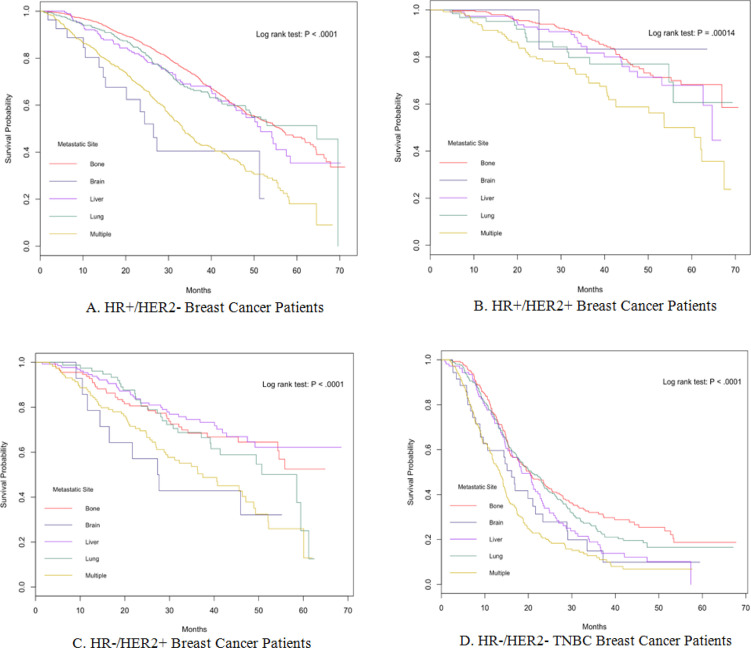
Kaplan-Meier Curves of Overall Survival Comparing Patients with Metastases to Different Organs in HR+/HER2- (A), HR+/HER2+ (B), HR-/HER2+ (C) and TNBC (D)

Log rank tests and univariate Cox models stratified by subtype showed metastatic site significantly affected OS (HR+/HER2+ subtype: P = 0.00014; other subtypes: P < 0.0001, Figure 1). In HR+/HER2- breast cancer, the overall survival difference in patients with multiple metastases and brain metastasis was non-significant (P = 0.219, Table 3). Similarly, the overall survival difference with metastasis to liver, lung and bone was non-significant (Liver vs. Lung: P = 0.241; Bone vs. Liver: P = 0.055; Bone vs. Lung: P = 0.180, Table 3). The overall survival with multiple metastases or brain metastasis was significantly worse than that with metastasis to lung, liver or bone (Bone vs. Brain, Liver vs. Multiple, Bone vs. Multiple, Lung vs. Multiple: P < 0.0001; Brain vs. Liver: P = 0.002; Brain vs. Lung: P = 0.0004, Table 3). 

In HR+/HER2+ subtype, the only significant worse overall survival was when patients had multiple metastases (Bone vs. Multiple: P < 0.0001; Liver vs. Multiple: P = 0.004; Lung vs. Multiple: P = 0.018). No significant difference was observed in patients with metastasis to bone, liver, lung or brain (Bone vs. Brain: P = 0.333; Bone vs. Liver: P = 0.178; Bone vs. Lung: P = 0.203; Brain vs. Liver: P = 0.260; Brain vs. Lung: P = 0.225; Liver vs. Lung: P = 0.47, Table 3).

In HR-/HER2+ subtype, the overall survival was very similar with that in HR+/HER2- subtype. The overall survival with multiple metastases or brain metastasis was significantly worse than that with metastasis to lung, liver or bone (Bone vs. Brain: P = 0.012; Brain vs. Liver: P = 0.003; Brain vs. Lung: P = 0.045; Bone vs. Multiple: P = 0.001; Liver vs. Multiple: P < 0.0001; Lung vs. Multiple: P = 0.013, Table 3). The overall survival difference was non-significant when comparing patients with metastasis to bone, liver or lung (Bone vs. Liver: P = .191; Bone vs. Lung: P = .217; Liver vs. Lung: P = .053). 

In TNBC, patients with brain metastasis or multiple metastases had the worse overall survival (Bone vs. Brain: P = 0.009; Brain vs. Lung: P = 0.032; Bone vs. Multiple, Lung vs. Multiple: P < .0001; Liver vs. Multiple: P = 0.001). Patients with bone metastasis had significant worse overall survival than those with metastasis to brain or liver (Bone vs. Brain: P = .009, Bone vs. Liver: P = 0.011). The difference of overall survival in patients with metastasis to bone or lung was non-significant (P = 0.154, Table 3). 

*Breast cancer patients with bone oligometastasis had significant worse survival than patients without metastasis in all subtypes*

**Figure 2:**
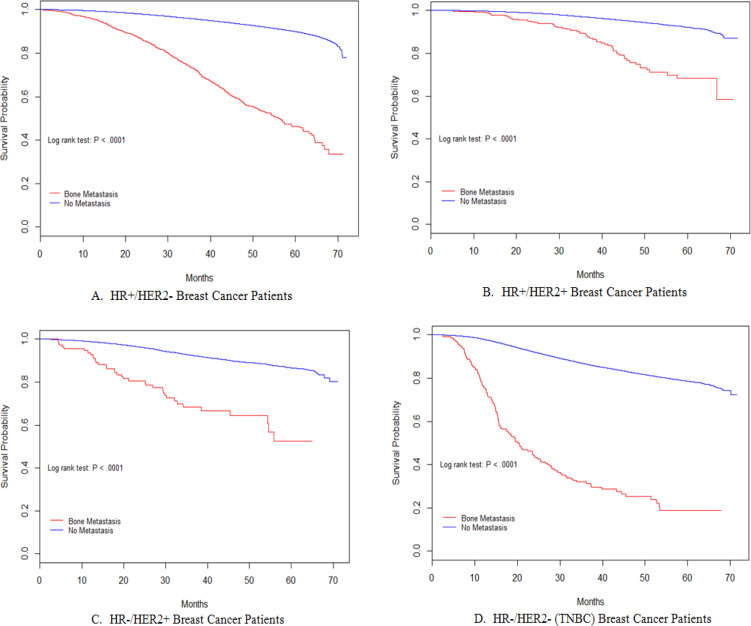
Kaplan-Meier Curves of Overall Survival Comparing Patients with Bone Oligometastasis and Patients without Any Metastasis in HR+/HER2- (A), HR+/HER2+ (B), HR-/HER2+ (C) and TNBC (D)

Among patients with metastatic disease, those with bone metastasis tend to have better overall survival (Table 3). We further compared the overall survival of patients with bone oligometastasis and patients without any metastasis. Patients with bone oligometastasis had significantly worse overall survival than patients without any metastasis in every breast cancer subtype (all P < .0001, Table 4) (Figure 2).

**Table 1:** Characteristics of NCDB Breast Cancer Ppatients Diagnosed from 2010 to 2013

Characteristic	Number of Patients (%)
Age at Diagnosis (n = 414,528)	
≤ 50	97,090 (23.42)
> 50	317,438 (76.58)
Chemotherapy (n = 372,484)	
Yes	198,051 (53.17)
No	174,433 (46.83)
Radiation Therapy (n = 412,697)	
Yes	272,705 (66.08)
No	139,992 (33.92)
Hormonal Therapy (n = 406,886)	
Yes	353,152 (86.79)
No	53,734 (13.21)
HER2 Targeted Therapy (n = 412,999)	
Yes	11,706 (2.83)
No	401,293 (97.17)

Patients might have received more than one therapy

**Table 2:** Distribution of Metastatic Sites in Each Subtype

Subtype	Metastatic Site					
	Single Metastasis				Multiple Metastases	No Metastasis
	Bone	Brain	Liver	Lung		
HR+/HER2-	2144	27	194	335	556	320803
(n = 324059)	(0.66)	(0.01)	(0.06)	(0.1)	(0.17)	(99)
HR+/HER2+	304	7	113	63	130	28054
(n = 28671)	(1.06)	(0.02)	(0.39)	(0.22)	(0.45)	(97.85)
HR-/HER2+	112	14	131	80	116	14181
(n = 14634)	(0.77)	(0.1)	(0.9)	(0.55)	(0.79)	(96.9)
TNBC	243	35	106	257	218	46305
(n = 47164)	(0.52)	(0.07)	(0.22)	(0.54)	(0.46)	(98.18)

The numbers were expressed as absolute number (%); P < 0.0001 in Chi-square test

**Table 3:** Univariate Analysis for Overall Survival Comparing Different Sites of Metastasis in Different Subtypes

Metastatic Site	HR+/HER2-		HR+/HER2+		HR-/HER2+		TNBC	
	Hazard Ratio (95% CI)	P	Hazard Ratio (95% CI)	P	Hazard Ratio (95% CI)	P	Hazard Ratio (95% CI)	P
Bone vs. Brain	0.35 (0.21-0.60)	<0.0001*	1.55 (0.21-11.17)	0.333	0.43 (0.21-0.90)	0.012*	0.62 (0.41-0.92)	0.009*
Bone vs. Liver	0.82 (0.64-1.05)	0.055	0.80 (0.50-1.28)	0.178	1.23 (0.77-1.95)	0.191	0.74 (0.57-0.96)	0.011*
Bone vs. Lung	0.91 (0.75-1.11)	0.18	0.78 (0.43-1.40)	0.203	0.82 (0.51-1.34)	0.217	0.90 (0.73-1.11)	0.154
Bone vs. Multiple	0.44 (0.38-0.50)	<0.0001*	0.41 (0.28-0.61)	<0.0001*	0.50 (0.33-0.76)	0.001*	0.49 (0.40-0.61)	<0.0001*
Brain vs. Liver	2.32 (1.31-4.12)	0.002*	0.52 (0.07-3.83)	0.26	2.85 (1.37-5.92)	0.003*	1.20 (0.78-1.84)	0.203
Brain vs. Lung	2.58 (1.48-4.50)	0.0004*	0.51 (0.07-3.84)	0.255	1.91 (0.90-4.02)	0.045*	1.46 (0.98-2.17)	0.032*
Brain vs. Multiple	1.24 (0.72-2.11)	0.219	0.27 (0.04-1.94)	0.096	1.16 (0.57-2.33)	0.341	0.80 (0.54-1.19)	0.131
Liver vs. Lung	1.11 (0.83-1.50)	0.241	0.98 (0.51-1.88)	0.47	0.67 (0.41-1.09)	0.053	1.22 (0.94-1.57)	0.066
Liver vs. Multiple	0.53 (0.41-0.69)	<0.0001*	0.52 (0.32-0.84)	0.004*	0.41 (0.27-0.62)	<0.0001*	0.67 (0.51-0.86)	0.001*
Lung vs. Multiple	0.48 (0.39-0.59)	<0.0001*	0.53 (0.29-0.96)	0.018*	0.61 (0.39-0.94)	0.013*	0.55 (0.45-0.67)	<0.0001*

* Significant P value (<0.05)

**Table 4:** Univariate Analysis for Overall Survival Comparing Patients with Bone Oligometastasis vs Patients without Any Metastasis

Subtype	Hazard Ratio (95% CI)	P
HR+/HER2-	7.50 (6.95-8.09)	< 0.0001*
HR+/HER2+	4.61 (3.52-6.03)	< 0.0001*
HR-/HER2+	4.73 (3.40-6.59)	< 0.0001*
TNBC	8.85 (7.58-10.34)	< 0.0001*

P values were for log-rank tests; * Significant P value (<0.05)

## Discussion

Breast cancer outcomes are related to the tumor biology, specifically the ability of a tumor to metastasize to distant sites and potentially lead to death. We previously studied a large database and we showed that breast cancer subtypes have different overall survival and breast cancer specific survival (Li et al., 2017; Li et al., 2018). In this study, we examined the relationship between breast cancer subtypes and the propensity for metastasis (both rate and location) and overall survival. 

The early work of Stephen Paget noted that metastasis in breast cancer was not by chance (Paget, 1889). He proposed that there was a relationship between the “seed” (breast cancer cell) and the “soil” (organ/site of metastasis). A century later, Perou et al., (2000) detailed the “molecular portrait” of breast cancer and subtyped breast cancer based on gene expression patterns. Further studies showed HER2+ and TNBC tumors had worse OS and disease free survival (Li et al., 2017; Li et al., 2018; Sorlie et al., 2001; Sorlie et al., 2003). Breast cancer subtypes also exhibit different response to chemotherapy, with HER2+ and basal-like TNBC having higher response rates to cytotoxic agents (Krishnamurti et al., 2017; Li et al., 2017; Li et al., 2016; Li et al., 2016; Rouzier et al., 2005; Wright et al., 2017). Thus, knowledge in the pattern of disease spread in breast cancer subtypes is important not only for treatment and surveillance, but also for patient and clinician expectations.

This study showed metastatic site affects OS. In all subtypes, brain metastasis and metastasis to multiple sites were correlated with the shortest OS. As expected, patients with bone only metastasis had the highest OS. It is important to note that OS was impacted by the presence of any metastasis regardless of the site and extent. Patients with oligometastatic bone disease had significantly lower OS than patients without any distant disease. Interestingly, this decrement in OS was different between subtypes. TNBC patients with bone oligometastasis experienced the lowest OS while HER2+ patients had the highest. Thus, even with the same site of metastasis, the cancer subtype contributed to patient mortality. 

Although not with highest rate of metastasis in this study, TNBC patients had the poorest survival, which is consistent with previous studies (Garcia Fernandez et al., 2012; Gerratana et al., 2015; Gong et al., 2017; Kast et al., 2015; Vona-Davis et al., 2014). Such poor survival is likely related to aggressive tumor biology. Other studies examined patients with brain metastasis, and showed that TNBC patients with brain only metastasis had a lower survival than patients with other subtypes (Rostami et al., 2016). Similarly, in patients with bone only metastasis, TNBC again had a lower OS than other subtypes. This strongly indicates that not only the site of metastasis, but also the subtype of the breast cancer determines outcomes. Lin, et al., examined the NCCN database and noted that when controlling for confounding factors including site of metastasis, TNBC patients with metastatic disease had poorer outcomes (Lin et al., 2012). Lung was the most common site of metastasis in TNBC, a finding that was consistent with other studies (Gerratana et al., 2015; Wang, 2017; Wu, 2017).

This study showed HER2+ cancer had a higher rate of metastasis than HER2- cancer, regardless of HR status. In a closer look, patients with HR-/HER2+ disease showed a very different pattern of metastasis and OS compared with patients with HR+/HER2+ disease. Patients with HR-/HER2+ tumor had a markedly higher rate of metastasis to brain, liver and lung, while the HR+/HER2+ had higher bone metastasis. These findings are consistent with other reports whereby the HR+ cohorts had a longer OR than the HR- cohort (Gerratana et al., 2015). In two separate studies examining the SEER database, the HR-/HER2+ patients had higher rates of brain and liver metastasis as well as lower OS when compared with the HR+/HER2- patients (Wang, 2017; Wu, 2017). HR+/HER2- cancer had the highest rate of bone metastasis. HR+/HER2- cancer patients with multiple metastases had a poorer OS compared with patients with lung, liver or bone metastasis. These results are consistent with previously published reports(Garcia Fernandez et al., 2012; Gerratana et al., 2015; Kennecke et al., 2010; Savci-Heijink et al., 2015) 

The differential patterns of metastasis and survival in different breast cancer subtypes are likely due to different gene and protein expression profiles. Sihto, et al found similar results to ours, with luminal A preferentially metastasized to bone, HER2+ to lung and liver, and TNBC to liver and brain (Sihto et al., 2011). On further analysis, they noted that protein expression differed based on the preferential site of metastasis. Cancer with bone metastasis showed estrogen receptor and SNAI1 over-expression; cancer with liver metastasis showed SNAI1 over-expression; and cancer with lung metastasis showed EGFR, CK5 and HER2 protein over-expression (Sihto et al., 2011). In another study, Smid and colleagues examined tissue microarrays and showed the presence or absence of the WNT/beta-caterin pathway was related to brain or bone metastasis, respectively (Smid et al., 2008). They also found a possible role of adhesion molecules in lung metastasis. In addition, they found different pathways related to bone metastasis were correlated with ER and HER2 status. Therefore, these and our studies strongly indicate breast cancer subtypes have different metastatic pattern and prognosis.

The overall rates of metastasis were lower in this study compared with published reports (Wu, 2017). It is possible that the lower rate was due to our strict inclusion criteria with the inclusion of only patients who received surgery and systemic therapy. Patients with inoperable metastatic breast cancer might not undergone surgical management thus would not have been included in this cohort (Bafford et al., 2009; Blanchardet al., 2008). In the NCBD from 2010-2013, there were 4,252 patients who received at least one systemic therapy (hormonal, chemo- or anti-HER2 therapy) but did not have recorded surgery. Among these 4,252 patients, 1,428 had bone metastasis; 36 had brain metastasis; 234 had liver metastasis; 276 had lung metastasis and 1,193 had multiple metastases. Since it is unclear whether no record of surgery was because of inoperable disease or under documentation or under treatment, these patients were not included in this study. In addition, the metastatic pattern is similar to the pattern in this study and the total metastatic events in these 4,252 patients accounted for <1% of the total patient population included in this study. Therefore, the conclusions would not change even including these patients. Another possibility was that patients with seemingly low stage disease might not undergo a metastatic survey. 

There are a few limitations of this study. The first limitation was the follow-up time was relatively short with a median follow-up of just over 36 months. This is especially important for those patients with HR+ breast cancer, who would have late metastatic events. The second limitation was that while 10.45% of the patients had HER2+, only 2.83% received anti-HER2 therapy. This means that 72.97% of the HER2+ patients did not receive anti-HER2 therapy, which may have led to a higher rate of metastasis. This low treatment rate may be an issue of reporting than actual treatment, and likely would not have an impact on site of metastasis. For example, if the anti-HER2 therapy was given at an outpatient facility, it might not be recorded by the NCDB.

In conclusion, this is one of the largest studies to date examining the relationship between breast cancer subtype with rate and site of metastasis and outcomes in secondary metastatic breast cancer. We showed different subtypes have different metastatic patterns. The OS was not only associated with metastasis but also associated with breast cancer subtype. This study would help clinicians to better identify patients at risk of metastasis and tailor treatment according to breast cancer subtype.
